# Hemoporfin-mediated photodynamic therapy on normal vasculature: implications for phototherapy of port-wine stain birthmarks

**DOI:** 10.18053/jctres.02.201603.003

**Published:** 2016-09-03

**Authors:** Wesley J Moy, Gang Ma, Kristen M Kelly, Bernard Choi

**Affiliations:** 1 *Beckman Laser Institute and Medical Clinic, University of California, Irvine, CA, United States*; 2 *Department of Biomedical Engineering, University of California, Irvine, CA, Unites States*; 3 *Department of Surgery, Shanghai Ninth People s Hospital, Shanghai, China*; 4 *Department of Dermatology, University of California, Irvine, CA, United States*; 5 *Department of Surgery, University of California, Irvine, CA, United States*; 6 *Edwards Lifesciences Center for Advanced Cardiovascular Technology, University of California, Irvine, CA, United States*

**Keywords:** photochemical, photothermal, talaporfin sodium, benzoporphyrin derivative monoacid ring A, skinfold, laser speckle contrast imaging, laser speckle contrast analysis

## Abstract

**Background:** Port-wine stain (PWS) birthmarks currently are treated using a pulsed dye laser (PDL) combined with transient cooling of the epidermis. PDL treatment protocols utilize short pulses of light (585 or 595 run wavelength) to heat selectively the microvasculature due to absorption by intravascular hemoglobin. Although most patients respond to PDL therapy, few experience complete removal of the PWS. An alternate treatment option to PDL therapy of PWS is photodynamic therapy (PDT). Research groups have reported on various photosensitizers for PDT of PWS, including Hemoporfin, Benzoporphyrin Derivative monoacid ring A, and talaporfin sodium.

**Aim:** Our aim was to evaluate, with an established preclinical *in-vivo* model, the efficacy of photodynamic therapy (PDT) with Hemoporfin to achieve persistent vascular shutdown.

**Methods:** To monitor the microvasculature, a dorsal window chamber was surgically installed on 24 adult mice. The PDT excitation source emitted 150mW of 532nm light, with an irradiance of 100mW/cm^2^. A retroorbital inj ection of Hemoporfin (2 mg/kg) was performed to deliver the drug into the bloodstream. Laser irradiation was initiated immediately after injection. To monitor blood-flow dynamics in response to PDT, we used laser speckle imaging. We employed a dose-response experimental design to study the efficacy of Hemoporfin-mediated PDT to achieve persistent vascular shutdown observed on Day 7 after PDT.

**Results:** We observed four general hemodynamic responses to PDT: (1) At low radiant exposures, we did not observe any persistent vascular shutdown; (2) at intermediate radiant exposures, we observed delayed vascular shutdown effect with significant change to the vascular structure; (3) at intermediate radiant exposures, we observed an acute vascular shutdown effect with gradual restoration of blood flow and no significant changes to the vascular structure; and (4) at high radiant exposures, we observed acute vascular shutdown that persisted during the entire 7-day monitoring period, with no change in vascular structure. With light dose-response analysis, we estimated a characteristic radiant exposure of 359 J/cm^2^ that was required to achieve persistent vascular shutdown observed on Day 7 after PDT.

**Conclusions:** The experimental data collectively suggest that Hemoporfin-mediated PDT can achieve persistent vascular shutdown of normal microvasculature. However, compared with our previous data using Talaporfin Sodium as photosensitizer, Hemoporfin-mediated PDT is less efficient and requires a considerably longer (~four times) irradiation time.

**Relevance for patients:** Patients with PWS lesions may benefit from the advantages that PDT potentially offers over conventional PDL therapy. PDT potentially is safer for patients of all skin types and more effective at treatment of recalcitrant lesions. Although clinical data suggest that Hemoporfin-mediated PDT is a promising alternative to PDL therapy, our results suggest that additional study of other photosensitizers is warranted.

## 1. Introduction

Port wine stain (PWS) birthmarks are congenital vascular malformations that are typically found on the face and neck regions [1]. Current treatment protocols in the United States involve the use of a pulsed dye laser (PDL) combined with cryogenic cooling of the skin [2]. Yellow light, in the 585-595 nm wavelength range, is strongly absorbed by intravascular hemoglobin and can photocoagulate the targeted vasculature. Unfortunately, light in this spectral range is also strongly absorbed by epidermal melanin. This competitive absorption limits the light available for absorption by the targeted vasculature, and hence limits the efficacy of PDL treatment. To this end, a need exists to evaluate alternate approaches to treat PWS vasculature in a safe and effective manner.

An alternate treatment option to PDL therapy of PWS is photodynamic therapy (PDT). Optical excitation of a photosensitizer within the PWS blood vessels can result in specific targeted injury of the vessels via generation of cytotoxic singlet oxygen [3]. Today, PDT of PWS is most commonly performed in China, withPhotocarcinorin(PSD-007) [4] or Hemoporfin [5,6] typically used as the photosensitizer. In 2007, Gu *et al.* [4] reported on clinical observations collected from PDT of 1949 lesions in 1385 PWS patients. They identified 128 cases (6.6%) with complete clearance of the PWS, but also identified 145 lesions (7.4%) with clearance below 50%. Subjects treated with PDT were required to remain out of sunlight for four weeks. In 2011, Xiao *et al.* [6] published data from 642 patients collected over a five-year period, with an average of three to eight treatment sessions per person. They observed 5% of patients with complete clearance (100%), 30% of patients with more than 50% clearance, and over 70% of patients with more than 25% clearance. Ten percent of patients experienced complications including blistering, hypopigmentation, hyperpig-mentation, scarring, and photoallergy, over a time period of up to two months. Due to the long duration of photo sensitivity, patients were required to avoid sunlight for two weeks after PDT. Collectively, these results demonstrate the potential efficacy of PDT as a viable treatment approach for PWS. However, a prolonged photosensitivity period and a high risk of scarring are side effects that must be taken into consideration.

In previously published studies[7-10], Benzoporphyrin Derivative Monoacid Ring A (BPD) and Talaporfin sodium (TS) were investigated as photosensitizers for vascular-specific PDT and as components of a combined treatment protocol of photodynamic therapy and PDL irradiation (PDT+PDL) [11]. With PDT+PDL, the authors hypothesized that the two treatments could induce photocoagulation of PWS vessels via a synergistic effect, while simultaneously minimizing risks of scarring associated with each treatment, with the use of lower radiant exposures for both PDT and PDL. BPD was investigated because it has vascular specific properties and it is a FDA-approved clinical treatment for wet age-related macular degeneration [12]. In an *in-vivo* preclinical study, the efficacy of BPD-mediated PDT, PDL, and PDT+PDL protocols were compared over a five-day monitoring period. PDT+PDL was observed as the only treatment that consistently resulted in persistent vascular shutdown sustained over the entire monitoring period. In 2012, TS-mediated PDT was evaluated in an *in-vivo* preclinical study and estimated that a characteristic radiant exposure of 85 J/cm^2^ was necessary to achieve persistent vascular shutdown [8].

The objectives of the current study were to 1) establish a characteristic radiant exposure to achieve persistent vascular shutdown with Hemoporfin-mediated PDT, 2) compare the Hemoporfin-mediated PDT light dose required to achieve persistent vascular shutdown with that of NPe6-mediated PDT, and 3) determine the mechanism of action associated with Hemoporfin-mediated PDT.

## 2. Materials and Methods

### 2.1 Dorsal window chamber model

Similar to previous work [8,11,13], we utilized the mouse dorsal window chamber model installed on adult C3H mice (25-30 g, n = 24). The Institutional Animal Care and Use Committee at University of California, Irvine approved the *in vivo* experiments (IACUC protocol number 2002-2339).

### 2.2 Preparation of photosensitizer

Hemoporfin (Shanghai Fudan-Zhangjiang Bio-Pharmaceutical Company, Shanghai, China) was reconstituted with saline into a stock solution of 25 mg/mL. The drug dose (1 mg/kg) was selected based on clinical treatment parameters used in previous publications [4,5,6].

### 2.3 Laser irradiation

For Hemoporfin-mediated PDT, we utilized a diode-pumped solid-state laser (532 nm, Dragon Laser, Jilin, China) at an irradiance of 100 mW/cm^2^. We varied the irradiation time (0 to 5500 s) to achieve radiant exposures ranging between 0 and 550 J/cm^2^. A low irradiance was used to mitigate potential photo-thermal effects of the 532 nm excitation alone on the micro-vasculature.

### 2.4 Experimental design

We anesthetized and positioned the animal in a custom window chamber holder placed on top of a heating pad, identical to previously reported studies [8,11,13]. A mixture of isoflurane and oxygen was used as anesthesia. We administered Hemoporfin (2 mg/kg) via retro-orbital injection and initiated laser irradiation of the epidermal side of the window chamber immediately after injection. Irradiation times were randomized to minimize systematic bias. In total, we performed 24 experiments, with one experiment per cumulative radiant exposure.

### 2.5 Laser Speckle Imaging (LSI)

We used LSI to study blood-flow dynamics associated with Hemoporfin-mediated PDT irradiation. Briefly, a 633 nm Helium-Neon laser was used to illuminate the epidermal side of the window chamber and collected raw speckle image sequences from the dermal side. Using a simplified speckle imaging equation [14], we processed the collected images and calculated Speckle Flow Index (SFI) images. LSI methodology has been described previously in detail [13].

### 2.6 Dose response analysis

Similar to previous publications [8,11], we used the approach of dose-response analysis with a longitudinal study design. Raw speckle images were collected at the following time points: pre-PDT; immediately post-PDT; and at Days 1, 2, 3, and 7 after PDT. All authors independently reviewed the SFI images collected on Day 7 of each experiment and graded each image on a binary scale: a “0” (no persistent vascular shutdown, or incomplete vascular shutdown, on Day 7) or “1” (persistent vascular shutdown on Day 7) was assigned to each Day 7 image. We used a commercial software package (Prism version 5.0d, GraphPad Software, San Diego, CA) to apply a sigmoidal fit to the data and estimate a characteristic radiant exposure (RE_50_/7) at which persistent vascular shutdown was observed on Day 7 after PDT.

## 3. Results

**Figure 1.**
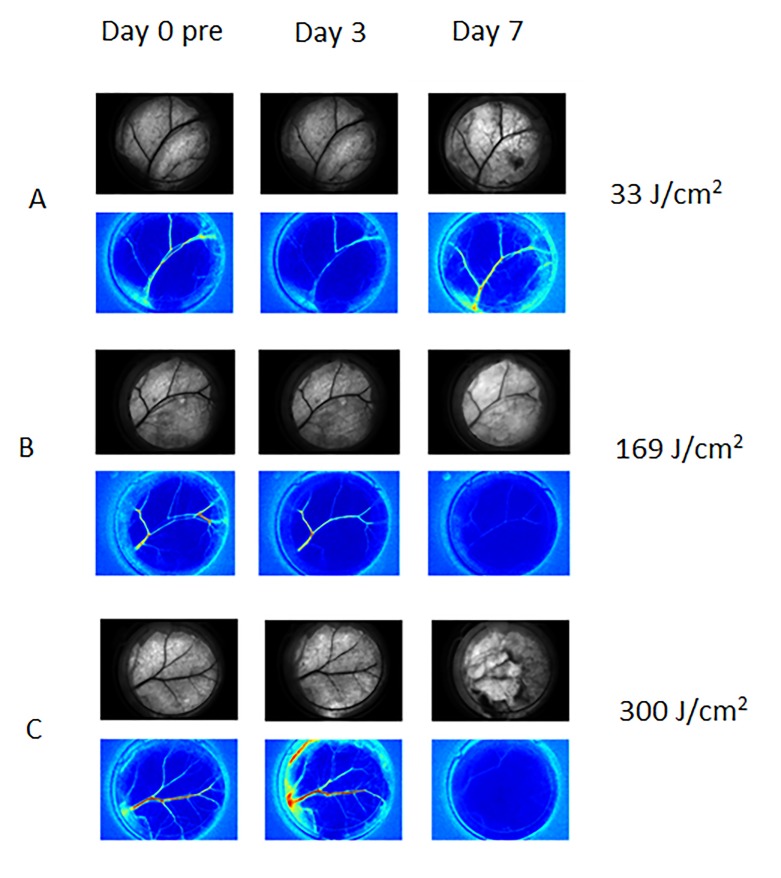
Representative Brightfield (top row of each set) and SFI images (bottom row of each set) collected from experiments involving Hemoporfin-mediated PDT, at Day 0 pre-PDT and Days 3 and Day 7 after PDT. A) With a sub-therapeutic radiant exposure of 33 J/cm^2^, we did not observe shutdown or reduction in flow, and vascular structure remained intact. B) At 169J/cm^2^, we observed a change in the morphological structure of the vasculature with complete shutdown observed on Day 7. C) At 300 J/cm^2^, we observed complete vascular shutdown with presence of hemorrhage formation.

**Figure 2.**
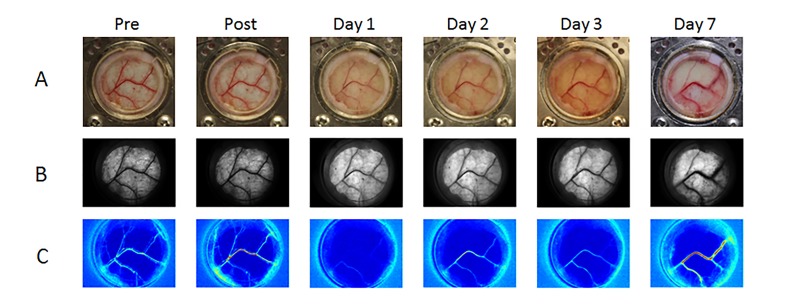
Representative example of Hemoporfin-mediated PDT using clinical treatment parameters (100 mW/cm^2^ irradiance, 2 mg/kg drug concentration, and 134 J/cm^2^ radiant exposure). We observed acute vascular shutdown, followed by gradual recovery of blood flow in the affected vasculature. We assigned a “0” score to this specific experiment, as there was evidence of blood flow recovery. A) Color and B) Brightfield images of the window chamber, indicating that blood vessel structure changed only minimally throughout the seven-day monitoring period. C) SFI images display persistence of functional flow within the blood vessels of the window chamber. Flow was reduced through Day 3 and was restored on Day 7.

Based on Brightfield images alone, the vascular architecture appeared unaltered; however, based on SFI images, the vessels did not contain flowing blood (Figures 1 and 2). Hemoporfin-mediated PDT caused shutdown in both large and small blood vessels, suggesting that it may be more effective in shutting down small-diameter PWS blood vessels that are not easily treated with the PDL.

To quantify the vascular effects of Hemoporfin-mediated PDT, we used LSI to map the microvascular response. We observed four general hemodynamic responses to Hemoporfin-mediated PDT (Figures 1 and 2). At radiant exposures in the range of 0-100J/cm^2^, we did not observe any persistent vascular shutdown or any change in vascular structure. At radiant exposures from 100 to 360 J/cm^2^, we either observed a delayed vascular shutdown effect, with observed change to the vascular structure; or an acute vascular shutdown effect with gradual restoration of flow, without any observed changes to the vascular structure (Figure 2). At radiant exposures greater than 360 J/cm^2^, we observed acute vascular shutdown that persisted over the seven-day monitoring period, without any change in vascular structure.

**Figure 3.**
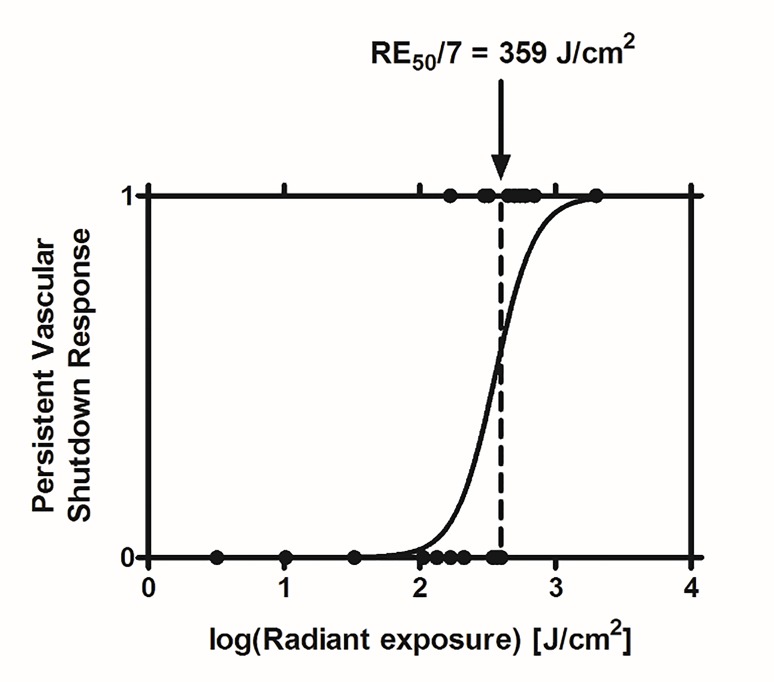
Dose-response curve of Hemoporfin-mediated PDT. Based on data from 24 experiments, we identified an RE_50_/7 of 359 J/cm^2^, associated with an irradiation time of ~60 min.

We utilized a previous grading system [8] to determine that 15 out of the 24 experiments did not result in persistent vascular shutdown at Day 7, while the remaining nine experiments resulted in persistent vascular shutdown at Day 7. We observed a clearly defined relationship between increasing radiant exposure and the expected result of persistent vascular shutdown. With dose-response analysis, we estimated an RE_50_/7 radiant exposure of 359 J/cm^2^ (95% confidence interval: 252-505 J/cm^2^) for Hemoporfin-mediated PDT (Figure 3).

## 4. Discussion

The experimental data collectively suggest that Hemoporfin-mediated PDT can achieve persistent vascular shutdown at a characteristic light dose of 359 J/cm^2^, using methodology similar to previous published clinical studies [5,15,16]. At high radiant exposures (> 360 J/cm^2^), Hemoporfin-mediated PDT consistently induced persistent vascular shutdown. In our previous studies [8,11] involving NPe6-mediated PDT, we identified a characteristic radiant exposure of 85 J/cm^2^ to achieve persistent vascular shutdown, which was equivalent to an irradiation time of lOmin, at the same 1 mg/kg drug dosage per mouse. In comparison, the characteristic radiant exposure of 359 J/cm^2^ associated with Hemoporfin-mediated PDT is equivalent to a treatment time of 60 min, which may be too long for patients to tolerate in the clinic.

Similar to NPe6-mediated PDT, we observed with Hemoporfin-mediated PDT acute vascular shutdown in which blood flow was shut down immediately after treatment but gradually returned by Day 7. A difference between NPe6- mediated and Hemoporfin-mediated PDT is that the latter resulted in minimal alteration of microvascular architecture, even when blood-flow shutdown was observed. With an intact microvascular structure, the biological response after Hemoporfin-mediated PDT may lead to more rapid vascular remodeling and repair, thus leading to a higher characteristic radiant exposure for photocoagulation than for NPe6-mediated PDT.

Previous studies of Hemoporfin-mediated PDT collectively suggest that it is an effective treatment for PWS. However, the reported photosensitivity period is four to eight weeks, with adverse effects of scarring, skin necrosis, and photothermal injury reported [4,15]. In our experiments, we observed shutdown of both smaller and larger vasculature in the irradiated region. Unfortunately, complete vascular shutdown within a tissue region may also result in skin necrosis, highlighting the need for additional studies on both safety and efficacy of Hemoporfin-mediated PDT.

In conclusion, our data collected during Hemoporfin-mediated PDT demonstrate that persistent vascular shutdown can be achieved, but only at a characteristic radiant exposure approximately four times greater than that required with NPe6-mediated PDT [8]. The reduced efficacy may be due in part to the lack of disruption of the vascular architecture caused by Hemoporfin-mediated PDT. Future studies are warranted to elucidate the desired mechanisms of photoinjury to achieve persistent vascular shutdown. Additional work can also include a combined protocol with PDL treatments, where Hemoporfin-mediated PDT is first performed, followed by PDL irradiation, similar to previous studies we have performed with NPe6-mediated PDT [11]. Hemoporfin-mediated PDT can achieve persistent vascular shutdown of normal microvasculature, but use of fairly high radiant exposures, or long treatments times, are required to achieve this effect.
